# Striatal Network Models of Huntington's Disease Dysfunction Phenotypes

**DOI:** 10.3389/fncom.2017.00070

**Published:** 2017-07-27

**Authors:** Pengsheng Zheng, James Kozloski

**Affiliations:** Computational Neuroscience and Multiscale Brain Modeling, Computational Biology Center, IBM Research Division, IBM T. J. Watson Research Center New York, NY, United States

**Keywords:** Huntington's disease, striatum, medium spiny neuron, network, dynamics, homeostatic plasticity, STDP, neurodegenerative

## Abstract

We present a network model of striatum, which generates “winnerless” dynamics typical for a network of sparse, unidirectionally connected inhibitory units. We observe that these dynamics, while interesting and a good match to normal striatal electrophysiological recordings, are fragile. Specifically, we find that randomly initialized networks often show dynamics more resembling “winner-take-all,” and relate this “unhealthy” model activity to dysfunctional physiological and anatomical phenotypes in the striatum of Huntington's disease animal models. We report plasticity as a potent mechanism to refine randomly initialized networks and create a healthy winnerless dynamic in our model, and we explore perturbations to a healthy network, modeled on changes observed in Huntington's disease, such as neuron cell death and increased bidirectional connectivity. We report the effect of these perturbations on the conversion risk of the network to an unhealthy state. Finally we discuss the relationship between structural and functional phenotypes observed at the level of simulated network dynamics as a promising means to model disease progression in different patient populations.

## 1. Introduction

Huntington's disease (HD) is an autosomal dominantly inherited neurodegenerative disease, which leads to widespread cell death in the striatum of humans who carry the mutant huntingtin gene. In particular principal cells of this structure known as medium spiny neurons (MSNs) die first. The mutant gene includes a translated trinucleotide repeat that encodes a poly-Glutamine (poly-Q) region of the Huntingtin protein, which when expanded beyond ~37 residues in the mutant form, guarantees the disease will emerge during an otherwise normal individual's life (Vonsattel and DiFiglia, 1998).

The neuronal structure affected most by HD, striatum, acts in global brain circuitry as the input stage to the Basal Ganglia (BG), a set of basal forebrain structures involved in behavioral reinforcement learning and action selection among all vertebrate organisms (Graybiel, 1998). GABAergic MSNs comprise ~90% of the tissue's neuronal population, such that the striatum's microcircuitry is almost entirely inhibitory, with collaterals of MSNs creating a sparsely connected network, free of bidirectional connections, and receiving excitatory drive from Layer 5 of neocortex (McGeorge and Faull, 1989; Czubayko and Plenz, 2002; Tunstall et al., 2002; Cepeda et al., 2013). Other inputs to MSNs derive from interneurons, including strong inhibition from a local feed-forward inhibitory network, which also receives Layer 5 inputs, and comprises fast-spiking, parvalbumin-positive interneurons (FSIs) (Gerfen et al., 1985; Luk and Sadikot, 2001; Taverna et al., 2007; Gittis et al., 2010; Berke, 2011; Szydlowski et al., 2013).

Experimental observations of the striatum have established that normal *in vivo* firing patterns of MSNs include episodic bursting activity, which appears spontaneously, even when the animal is at rest (Walker et al., 2008; Miller et al., 2011). Furthermore, these firing patterns seem inconsistent with activity observed in other networks that include lateral inhibition, such as sensory epithelia and neocortex. Here, receptive fields are sharpened and outputs made sparser by strong competition between neurons or neuronal groups (Sachdev et al., 2012).

### 1.1. Previous theoretical work

Theoretical work has made great progress in modeling the episodic bursting firing patterns of MSNs by carefully replicating distinctive properties of striatal microcircuitry in network models, such as sparse inhibitory connections and variable excitability among MSNs (Wickens et al., 1991; Angulo-Garcia et al., 2016). However, the importance of asymmetric connectivity in this network has been largely ignored.

Most recently, models of striatum have revisited the “winnerless” inhibitory network model, and derived from it a sophisticated network of biologically plausible units that show transitions in rate dynamics related to network stability and chaos (Ponzi and Wickens, 2010). Originally used to model an olfactory stimulus encoder in the locust, the winnerless network of Rabinovich et al. (2001) formalized intuitions about the requirements for episodic bursting in inhibitory networks, but in a much simpler model, which depends on asymmetric connectivity for these dynamics. Characterized by their ability to dynamically encode input patterns based on groups of neurons continuously competing through lateral inhibition, ongoing winnerless dynamics and network trajectories act as the output code. This coding is in contrast to more conventional “winner-take-all” network encoding schemes, which rely on lateral inhibition achieving a stable representational state as the network's output. Given that both networks depend on lateral inhibition for normal function, what makes the winnerless network's dynamics so different from winner-take-all?

First, winnerless network topology enforces an assymmetry by disallowing reciprocal connections between inhibitory units (Rabinovich et al., 2001). Winner-take-all networks instead rely on mostly reciprocal lateral connections between excitatory and inhibitory units to achieve stable representational states (Maass, 2000). Second, winnerless networks generate a constant total level of activity when their units are driven to spike. This activity results in the network continuously shaping its units' ongoing dynamics, even when inputs to those units are weak. In this way, winnerless networks encode a changing input rapidly as multiple units respond alternately, compete continuously for net excitatory drive, and implement a flexible population code. In contrast, winner-take-all networks, implemented with the same unit models, decrease their total activity during continued stimulation as initial competition settles and a few winners emerge. Escape from inhibition among silent non-winners is slow as inputs change, resulting in sparse, stable encoding by just a few constantly active units (Kohonen, 1982).

### 1.2. Current aims and approach

Given the complex dynamics present in a simple winnerless model of striatal activity, we aimed to validate this model against HD network-level phenotypic measures. In our previous work, we proposed a set of hypotheses surrounding neurodegenerative disease (Kozloski, 2016). In summary, network perturbations due to unary modifications to network parameters governing network properties such as signal propagation and plasticity were proposed as possible primary disease risks (stemming for example from the single gene mutation in HD). Our hypotheses included a proposed role for feedback and network dynamics in neural circuits to restore near-normal circuit set points, while at the same time increasing secondary risks of neuronal dysfunction, damage, or loss. We proposed that these secondary risks may lead to cascading failures or dysfunction of neural tissues as neuronal loss requires the accrual of even greater secondary risks to maintain normal network function.

Interestingly, well before MSNs die in HD model animals, they become dysfunctional (Cepeda et al., 2007; Estrada-Sanchez and Rebec, 2013), suggesting that altered signaling through corticostriatal circuitry may set the stage for HD and its subsequent progression. In support of this view, both cortical and striatal neurons show aberrant patterns of spiking activity in behaving R6/2 transgenic HD model mice (Walker et al., 2008; Miller et al., 2011). In particular, MSN firing patterns at times manifest sustained firing without the episodic bursting characteristics of normal striatal MSN firing (Miller et al., 2008) and the winnerless network. Striatal network connectivity is greatly disrupted in HD model animals, with the fraction of bidirectional connections between MSNs in R6/2 HD model mice greatly increased (Cepeda et al., 2013). Recent evidence also suggests altered communication between cortex and striatum in HD (Hong et al., 2012), and disturbances in both feed forward and, to a lesser extent, feedback inhibition to MSNs, which then drive increased GABAergic synaptic activity of indirect pathway MSNs in HD model mice (Cepeda et al., 2013).

The transition from winnerless dynamics to winner-take-all dynamics has been described previously, and occurred when network connectivity fractions and weights were increased (Ponzi and Wickens, 2013), although reciprocal connections were not explicitly removed from the network topology in this study. Here, we report how introduction of bidirectional connections into a wholly unidirectional network, as has been observed in the R6/2 HD model when compared to the wild-type (WT) background strain from which it was derived (Cepeda et al., 2013), may be of equal or greater importance to the transition from WT to HD network phenotypes. With this diseased network topology, the previous studies' transitions to winner-take-all dynamics might be altered, and we therefore emphasize in this study the importance of carefully controlling bidirectional connections to create a robust winnerless striatum model. We show how modeled disruptions can elucidate risks of phenotypic conversion and neurodegeneration in HD model organisms and humans. Finally, we consider how changes to normal maintenance and restorative mechanisms such as plasticity and balanced synaptic transmission are possible contributors to dysfunction.

### 1.3. Model overview

We employed a FitzHugh-Nagumo (FN) model derived from the work of Rabinovich et al. (2001) for all simulations of MSN dynamics in striatum. We reasoned that because of the robust winnerless-like dynamics exhibited by MSNs, and their similar asymmetric network topology, even a very simple model and basic network configuration may be sufficient to capture and study network phenotypes of the normal and dysfunctional striatum. Our base assumption is that certain striatal network dynamics are universal, given a set of simple network constraints (implementing winnerless vs. winner-take-all dynamics), and that network dysfunction may be similarly universal given modifications to these constraints. If this assumption is true, future work with more detailed models will remain constrained by the observations reported here for the simpler network model. (For example, even when using more physiologically detailed neurons and synapses in similar striatal microcircuit models of specific animal models of HD should yield results verifiable against our findings).

Briefly, a unit potential, recovery factor, and synaptic conductance were each modeled by a first-order ordinary differential equation (see Materials and Methods). Excitatory stimulation to units in the network was fixed at randomly chosen values with a small offset, in order to represent the inputs to striatum by a constant pattern of cortical activity in Layer 5. These inputs were varied during our simulations only where noted. We interpreted a single positive fluctuation in the unit potential (i.e., an FN spike) as a striatal MSN's burst of action potentials lasting ~350 ms. In Kozloski (2016), we termed these FN spikes the “burst potential” of the neuron, with a positive transient representing a 350 ms burst in the physiological neuron's spike train. This coarse resolution model of the neuron's membrane potential is adequate to capture the dominant bursting mode of firing in MSNs, and the observation that their activity is often alternating series of bursts of bursts (Miller et al., 2008).

Free parameters of our model were then the number of the neurons and their network connectivity for each simulation. Unless otherwise noted, the results reported here derived from simulations of a 500 neuron network. Similar qualitative results have also been verified for networks of size 50, 100, and 1, 000, though the magnitude of the effect given a perturbation may differ.

We show that changes such as (1) neuron silencing, which models cell death among MSNs, (2) disruptions in network connectivity (in particular to the bidirectional connection fraction), and (3) abnormal cortical inputs can each lead to HD phenotypes in our model. We further show how these disruption may be resolved or exacerbated by simple changes to the model's plasticity. Our approach creates a research path toward applying network models of striatal activity to therapeutic insights into HD.

## 2. Results

### 2.1. Criterion for healthy network dynamics

We present results indicating that HD neuron and circuit phenotypes may be modeled in a winnerless network model of striatum based on perturbations known to be associated with HD progression. As noted previously, *in vivo* recordings of MSNs display bursts that episodically recur throughout recorded spike trains in both WT and in behaving HD model mice (Miller et al., 2008). Furthermore, alternating bursts appear as the dominant feature of healthy MSN firing patterns in the WT background strain from which the R6/2 model was derived. Our model specifically addresses a localized population of WT MSNs, among which burst durations most often (defined as the middle 50 percentiles of all observations) occurred at 0.65−1.1 s durations (min < 0.1*s*, max ~ 4 s). Bursts occurred most often at a rate of ~ 1−4 per minute (max ~ 11), with 2−9% (max ~ 58%) of bursts occurring simultaneously across MSNs, and 12−54% (max ~ 90%) of all spikes occurring within bursts^1^.

**Figure 1:**
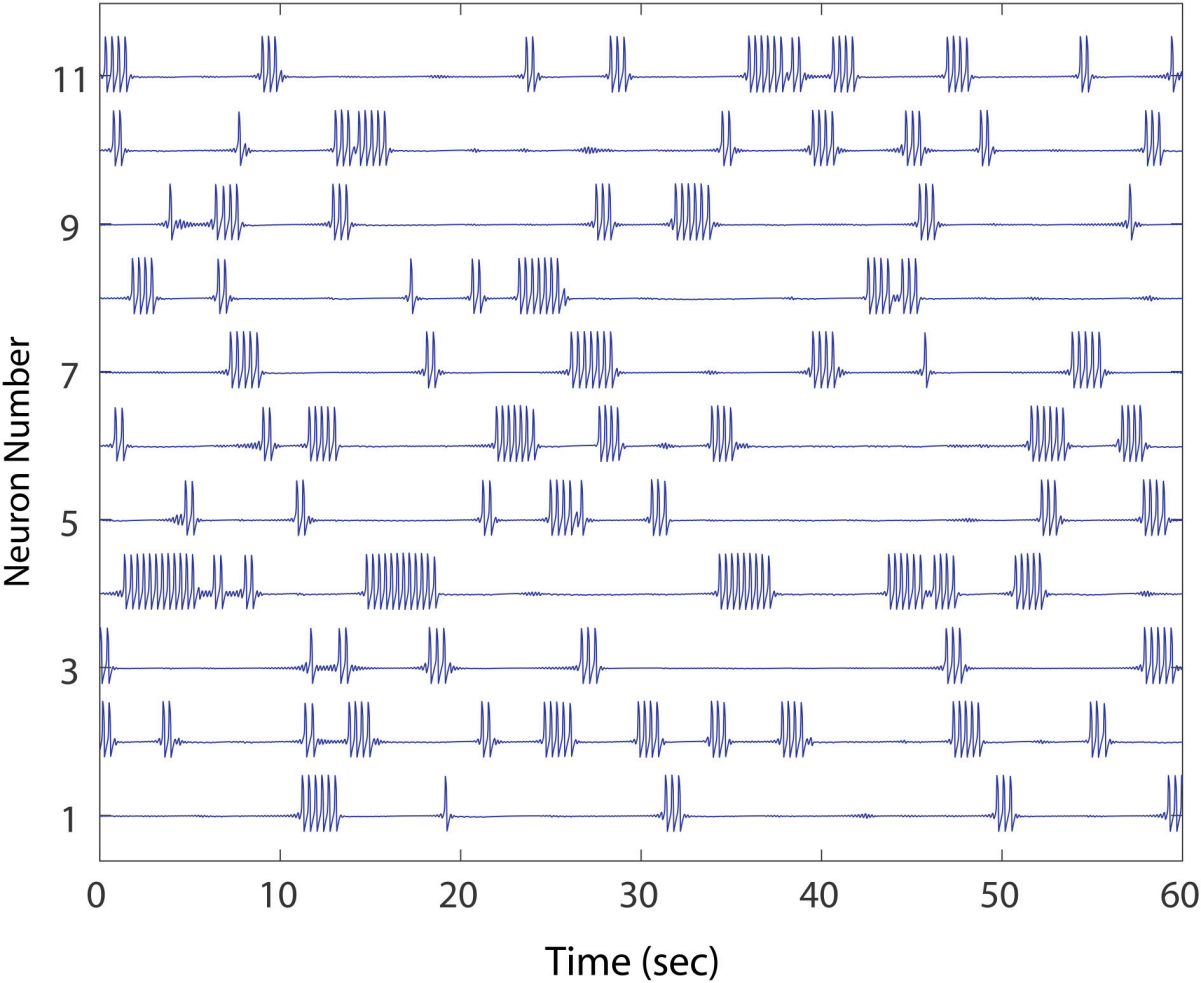
An example of healthy winnerless network dynamic. Shown are 11 out of the total 50 FN neuron network simulated. As in striatum, the network shows episodic “bursts of bursts” which are organized in neuronal activations that alternate, and which include no silent neurons. The network, derived from a preliminary evolution of a randomly initialized, unidirectional set of connections between neurons, meets our criteria for health.

Our model MSN deployed stereotyped FN spikes representing bursts of a short duration (~ 350 ms), which occurred at a higher rate in the winnerless network (~ 30 per minute) than in WT recordings. Furthermore, in our model, 100% of spiking activity occurred as bursting activity. We considered these deviations from the WT firing patterns acceptable, since the focus of our study was to address *network* burst dynamics and how winnerless network instability can drive units to cease firing in episodic bursts of bursts altogether (Figure 1), and instead fire continuously. In our study, the phenomenon of constant firing among FN model MSNs was robust under a variety of conditions, and we noted a similarity of this phenotype to those reported by Miller et al. (2008) (cf., Figure 9, R6/2 Units 1 and 5). Specifically, we defined normal bursting activity in our network as a period of continuous bursts (FN spikes) that falls within the range of burst durations observed (< 4 s), and at low rates of simultaneous bursting normally observed in WT animals. We further required that *all* units in the network burst at some point during 1 min of simulated stationary excitatory inputs in order for a network to be considered healthy (Figure 1), since at this initial condition, silencing of neurons was always correlated with the emergence of neurons that burst continuously (i.e., >4 s). These two criteria for a healthy network (1. episodic simultaneous bursting among *groups* of MSNs, and 2. episodic alternating bursting among *every* MSN) were then evaluated. To establish a baseline, we show different healthy networks (except where noted) for each experiment; the baseline was further confirmed for different healthy networks, not shown.

Our winnerless MSN network topology was modeled on that of Rabinovich et al. (2001), with a completely asymmetric set of connections and a connection fraction of 0.35 among unidirectionally connected pairs. We first noticed that randomly initializing such a network, subject to these constraints, was not sufficient to consistently produce healthy network dynamics. Specifically, by randomly initializing and simulating 100 distinct networks of size 500 units, we found that only ~ 30% of networks met the criterion that no unit should produce continuous bursting that exceeds the maximum burst duration of 4 s for networks of this size. Instead, in ~ 70% of networks, firing in at least one unit continued at a high rate for the duration of the simulation. Furthermore, among the 30% that showed normal firing rates, >90% failed to meet the criterion that every neuron burst at least once during a 1 min simulation. Silent neurons, which never fire regardless of the duration of the network simulation, occurred in almost all networks initialized in this manner, such that overall < 3% of randomly initialized networks were healthy.

### 2.2. Mechanisms to establish healthy dynamics

In order to study randomly initialized, asymmetric winnerless networks from a starting point of healthy dynamics, we aimed to first determine how to shape the network in order to establish a healthy state. Here we explore if mechanisms responsible for maintaining a physiologically balanced self-organizing recurrent network (SORN) (Zheng and Triesch, 2014) might also help balance activity in a winnerless network. Specifically, we studied the ability of intrinsic plasticity (IP) and inhibitory spike-timing dependent plasticity (iSTDP) to maintain network activity that satisfies our two criteria for healthy dynamics.

The IP mechanism described by Zheng and Triesch (2014) regulates spiking thresholds such that all neurons fire at the same rate. For the winnerless network, we chose a rate of bursts equal to the mean bursting rate of otherwise normally active MSNs (0.5/s). Spike detection in the FN neurons was performed on positive transients, and the FN thresholds of MSNs were not directly modified. Instead, by adding a spike-rate dependent intrinsic current, MSNs which would otherwise remain silent became active, and those which were constantly firing resumed episodic bursting.

Next, inhibitory weights in the asymmetrically initialized winnerless network were further refined by subjecting them to iSTDP. The iSTDP rule was derived from Zheng and Triesch (2014), and implements a logical weight modifier, such that if a unit is undergoing an FN spike, and the unit receiving inhibition from this unit stays silent for a period of time after the MSN bursts, the corresponding weight is weakened by a fixed amount. Thus, if an MSN succeeds in silencing a target MSN with its burst, its inhibitory influence on the postsynaptic MSN is weakened. Conversely, if the MSN fails to silence the postsynaptic MSN, its inhibitory influence on it is strengthened.

IP was sufficient to create a healthy network, according to our two criteria. Similar to IP, iSTDP was sufficient to restore healthy network dynamics to the MSN winnerless network. We observed that after iSTDP, the uniform distribution of initial weights had adopted a lognormal distribution. Furthermore, after modifying inhibitory weights between MSNs according to this rule, every unit became active at some point in the simulation. Furthermore, those units which were constantly firing resumed episodic bursting as inhibition from other units in the network was strengthened.

We did not attempt to combine these two mechanisms in a single simulation, however in SORNs we note the influence of each is complementary in establishing a normal network. For experiments with different network perturbations described below in different subsections, we established a single healthy asymmetric network using iSTDP and used it for all perturbation simulations.

### 2.3. Mechanisms leading to unhealthy dynamics

From these randomly initialized networks, balanced in their spike initiation by iSTDP, we next aimed to study perturbations to the networks that could disrupt these healthy dynamics and cause MSNs to enter a state of continuous firing. These studies were aimed at mimicking perturbations known to occur in HD.

Our initial perturbation study first established a healthy network dynamic, and then turned off iSTDP, thus using a fixed network topology without plasticity for perturbing the network and studying its failure in this vulnerable state. In real biological networks, iSTDP may remain constant and act as continuous compensating influence to counteract the sensitivity to perturbations we analyze here.

#### 2.3.1. Neuron silencing to model MSN cell death

We studied the effect of neuron silencing on the striatal winnerless network model dynamics. To silence a neuron in this study, it was sufficient to set the neuron's membrane potential, *x*(*t*), to zero resulting in elimination of its ability to influence network dynamics.

**Figure 2:**
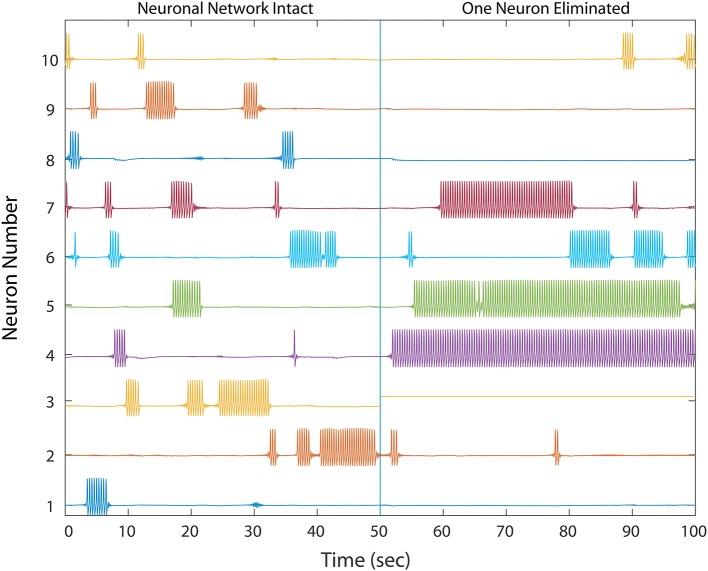
A healthy network shows winnerless activity (**left**) which abruptly changes when 1 neuron in a 50 neuron network is silenced, simulating neurodegeneration. Neurons which fire prolonged bursts are considered “unhealthy.” The network dynamics and trajectories shift to winner-take-all (**right**) in which some neurons fire continuously while others fall silent. In a simple three neuron motif, over-firing in unhealthy neurons inhibits downstream MSNs, producing silent “losers”, which fail in turn to provide proper inhibition to downstream neurons, thus producing a second winner. Multiple competitive interactions in this complex network makes the final outcome of the winner-take-all dynamics difficult to predict.

We first simulated cell silencing on a small 50 MSN network to gain better understanding of changes to dynamics. In several simulations, removing a single neuron from the network of size 50 was sufficient to transform it from a healthy to an unhealthy dynamics as shown in Figure 2. The winnerless pattern of firing relies on inhibition that a MSN receives from other neurons to transiently balance its constant excitation, and iSTDP was sufficient to establish this balance during initialization for all states that the network enters. In the absence of iSTDP, MSNs in the immediate downstream of the affected neuron suffered a permanent reduction in inhibitory input, and this change for some MSNs could destroy the balance and result in excess firing.

Excess firing among some MSNs then results in increased inhibition onto its immediate downstream neurons, which thereby destroys their balance, leading to decreased activity and even permanent silencing. We propose that this unhealthy dynamic continues to propagate, alternating between decreased and increased inhibition throughout the network to eventually cause the observed transformation from a winnerless network into a winner-take-all network. However, this propagation of unbalanced dynamics is almost impossible to analyze quantitatively in a complex network topology, which has many possible signal propagation paths for each MSN, such that even starting with those neurons responsible for the unhealthy dynamics results a complex global causal chain.

**Figure 3:**
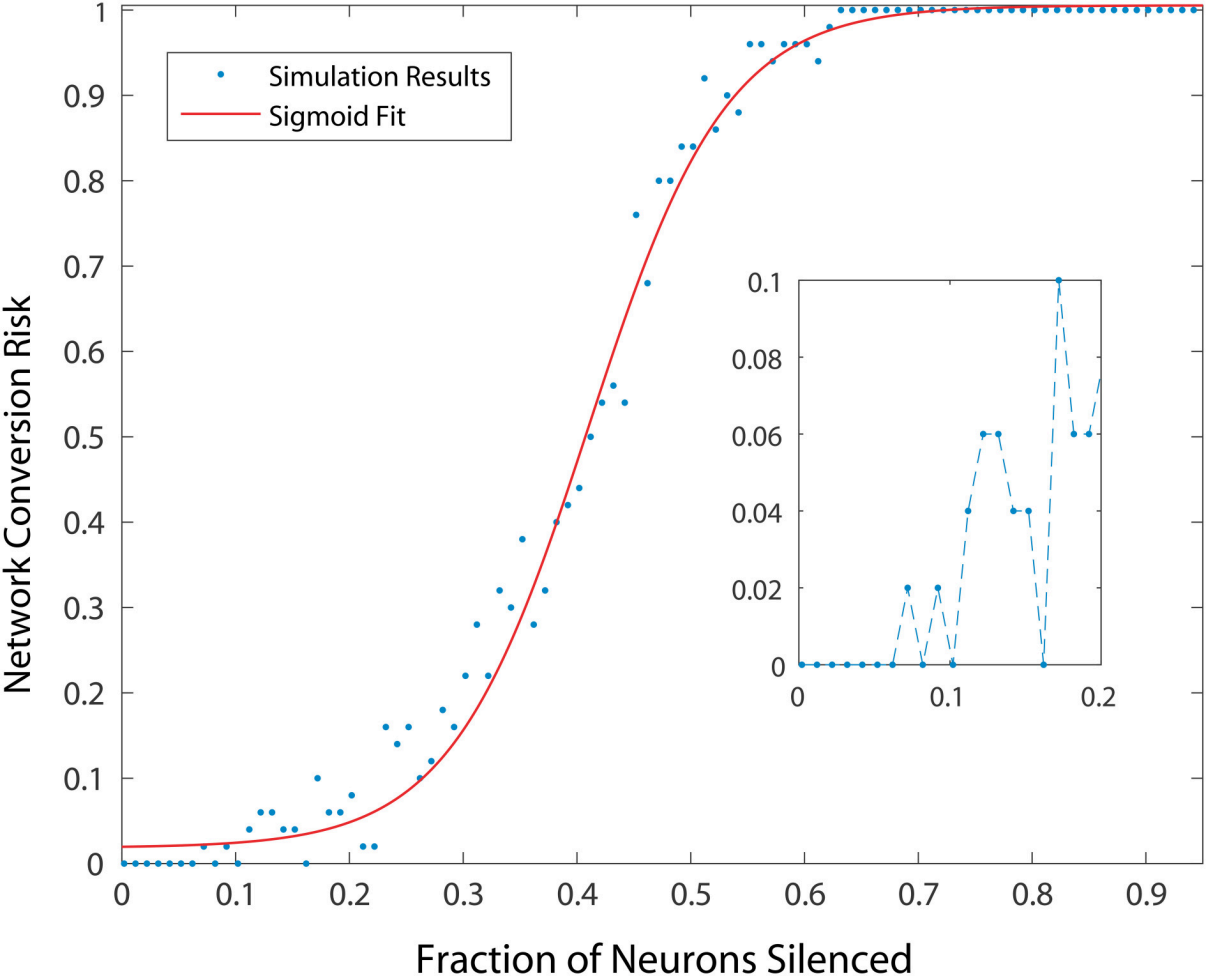
Probability of developing an unhealthy dynamic in at least one neuron in a 500 neuron network, as a function of fraction of neurons silenced (simulating neuron death in a real striatum). Network conversion risk is calculated using 20 simulations with different neurons silenced. For each data point the proportion of simulations in which an unhealthy neuron was observed is plotted. The red line represents a sigmoidal fit to the data, predicting a particular trajectory for disease progression.

To measure the effect of progressive neurodegeneration on network risk, we devised a means to measure the probability of transforming the network from healthy to unhealthy dynamics given a specific fraction of neurons silenced. A 500 neuron network was used, and for each network condition, 20 simulations were conducted. In each simulation, different populations were selected for silencing according to the specific fraction of cell silencing we aimed to model. From these 20 simulations, the probability of an unhealthy network was computed and the complex changes in network dynamics during different disease progression stages in the model was reduced to a single statistic. Figure 3 shows the probability of developing unhealthy dynamics as a function of neuron silencing fraction, and the function was well fitted by a sigmoidal.

#### 2.3.2. Bidirectional connectivity

As already noted, weights in our model network, *w*_*ij*_ were initialized in a randomly chosen asymmetric network topology and subjected to the iSTDP learning rule. The purpose of this weight initialization routine was to ensure that the network showed a healthy initial dynamics. Because iSTDP was turned off, we were able to perturb the same network topology in different ways in order to study how such changes might effect an unhealthy dynamic.

First, we examined how the introduction of bidirectional connections affects network dynamics. As previously noted, measurements from wild type mice, and presymptomatic R6/2 mice (Cepeda et al., 2013), indicate that bidirectional connections between MSNs do not exist, or are so rare that they were not observed in this study. In contrast, the rate of bidirectional connectivity in symptomatic R6/2 mice >30 days old is 50%. To study the clear progression to a high rate of bidirectional connectivity as the HD model phenotype progresses, we varied network bidirectional fraction, which is the percentage of the total number of connected pairs of neurons in the network that are bidirectionally connected. In addition, we varied the magnitude of inhibitory weights chosen for the new connections added to the already learned weights for the healthy network. For each of our ~ 50 bidirectional connection fraction to weight combinations, we ran 20 simulations to compute the probability of an unhealthy network for each combination, wherein each set of 20 is represented by a single probability, for a total of 1, 000 simulations.

**Figure 4:**
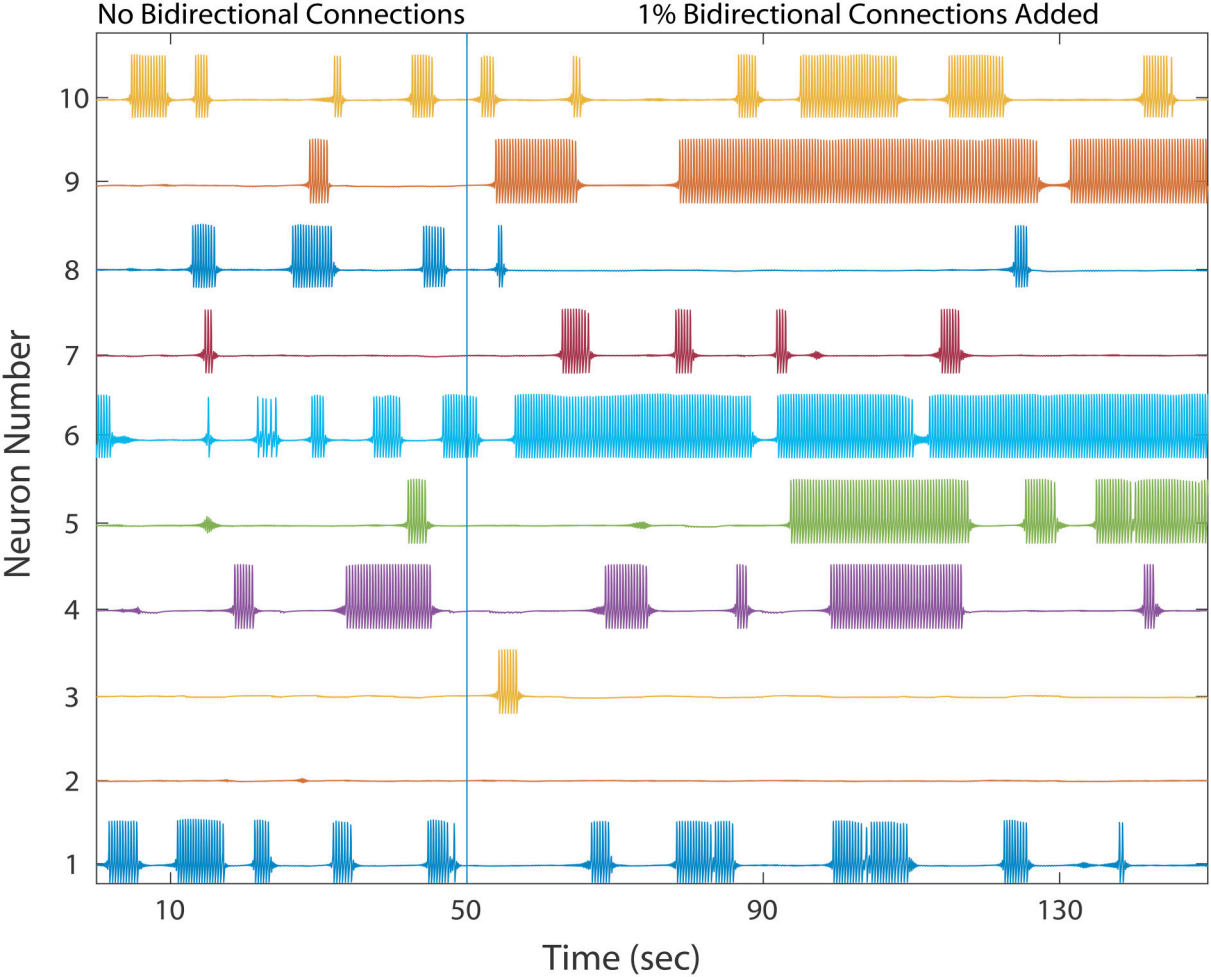
A healthy network again shows winnerless activity (left) which abruptly changes when a bidirectional connection is added to 1% of already connected neurons. 10 out of the 500 neurons are plotted. Bidirectional connectivity is observed to increase dramatically among MSNs in Huntington's disease model animals.

**Figure 5:**
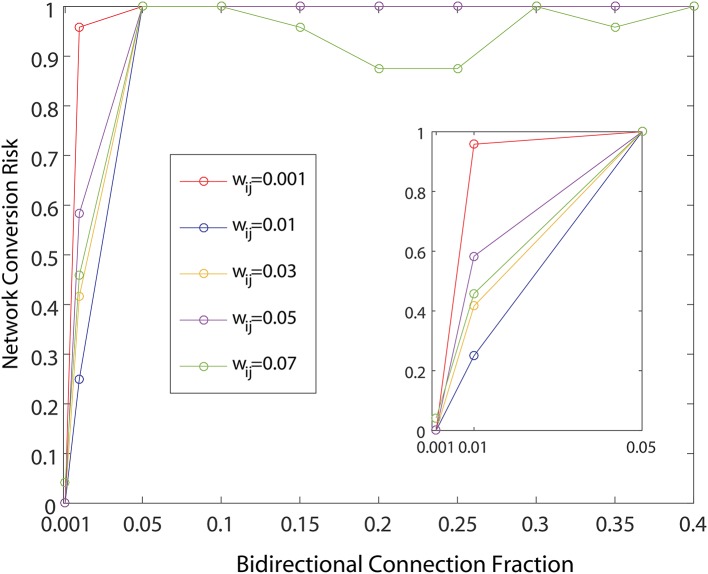
Probability of developing unhealthy dynamic in at least one neuron in a 500 neuron network simulation, as a function of the fraction of already connected neurons to which a bidirectional connection is added. For each curve a different weight for the bidirectional connection is used. All other network weights were developed by a preliminary refinement phase of the simulation using iSTDP. Network conversion risk is calculated as in Figure 3.

As shown in Figure 4, the network dynamics is sensitive to the introduction of bidirectional connections. The chosen weights of each added connection was relatively small, compared to those of the rest of the network, and therefore had only a modulatory effect on network dynamics. Furthermore, Figure 5 shows that after adding a fraction of bidirectional connections of just 1%, the probability of developing unhealthy dynamics in at least one neuron reached 0.98. This indicates the network is quite sensitive to this perturbation, and compensation must be robust to counteract this influence in the real HD striatum. Furthermore, the network inevitably showed an unhealthy phenotype if the bidirectional connection fraction grew larger than 5%. These results suggest a strong constraint on striatal microcircuitry to minimize risk, namely unidirectional connectivity, which is consistent with the experimental observation.

**Figure 6:**
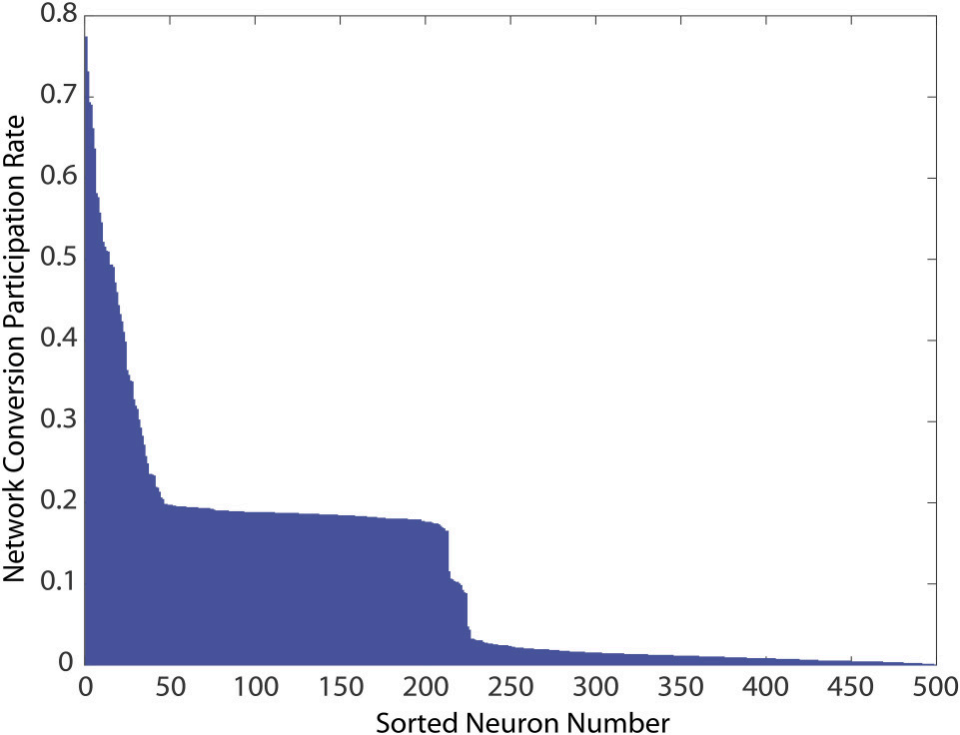
Network conversion risk by neuron index constructed using simulations from Figure 5. Because all simulations start from the same developed and healthy network, statistics for individually identified neurons can be pooled across simulations. For each simulation in which a network was classified unhealthy by at least one neuron showing unhealthy dynamics, neurons causing this classification due to prolonged firing were tabulated. The table was then used to construct the histogram showing their participation rate in causing the unhealthy network dynamics.

Finally, we explored if the specific neurons showing constant activity, and therefore responsible for the unhealthy network dynamics, were also topologically related to added bidirectional connections. Because all networks were initialized from the same starting configuration of weights, the identities of these unhealthy neurons were maintained across all 1, 000 simulations, and results could therefore be pooled according to neuron identity. We measured each neuron's risk to the network as the percentage of simulations in which it was responsible for identifying the network as unhealthy. As shown in Figure 6, in which these risk levels were ranked from high to low, the risk profile across neurons varies widely. First, a small portion of MSNs (neurons ranked 1–25) tended to consistently cause unhealthy dynamics no matter where in the network we added the bidirectional connection. We noted that the topological distance between the added connection, and the neuron was not predictive of conversion. This indicated that neurons were exposed to risks of conversion due to propagation of network dynamics, thereby suggesting a global cause to their unhealthy phenotype. Second, a broad plateau of risk of ~ 20% existed in the network for neurons ranked 50–200. Lastly, a large portion of MSNs (neurons ranked 250–500) tended to maintain healthy dynamics no matter where the network was perturbed.

#### 2.3.3. Abnormal cortical input

Because dysfunctional cortical inputs to the striatum have been implicated in the emergence of HD phenotypes in model animals (Estrada-Sanchez and Rebec, 2013), we aimed to examine if such dysfunction can also drive unhealthy network dynamics in our model. We observed that at higher input strengths, unhealthy network dynamics tended to develop, showing an abrupt transition when the randomly chosen excitatory input values, representing cortical inputs to our network, were suddenly increased above a threshold which we observed to vary depending upon the randomly initialized network topology and dynamics that derived from iSTDP conditioning. These results were consistent with those reported previously in studies of a winnerless network that included bidirectional connections (Ponzi and Wickens, 2012). We further observed that decreasing the inputs following this transition to unhealthy dynamics had a restorative effect on network health.

### 2.4. Mechanisms to restore healthy dynamics

#### 2.4.1. HD therapeutics

**Figure 7:**
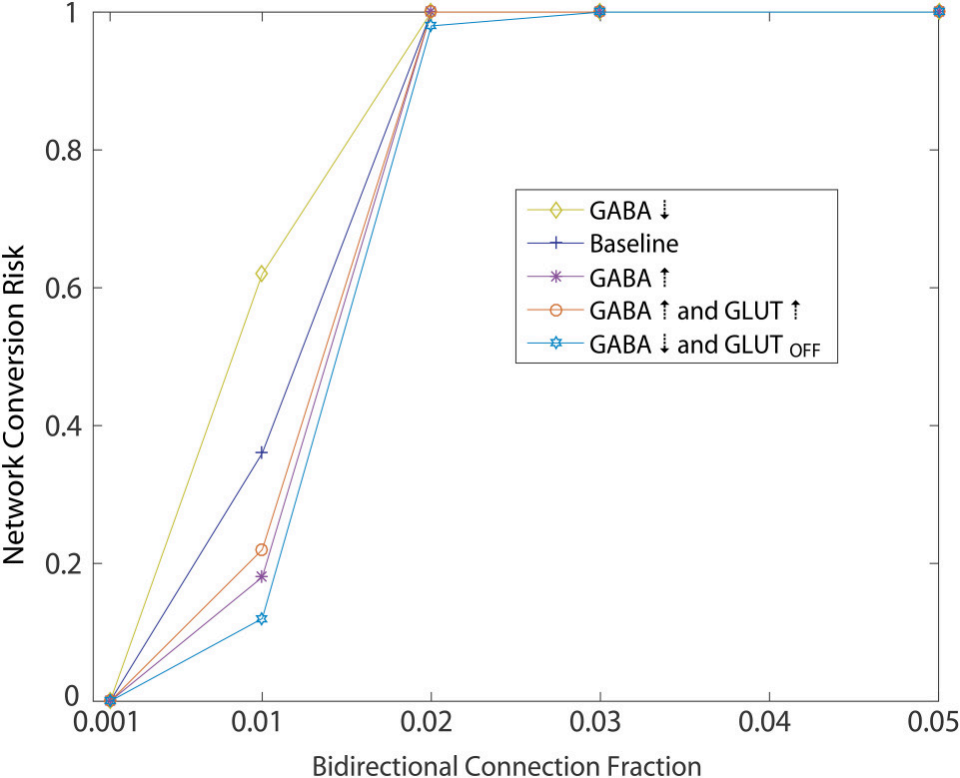
Simulated of HD therapeutics. Network conversion risk from Figure 5, *w*_*ij*_ = 0.001, plotted again in blue. Repeating the same simulation under different conditions aimed at modeling different secondary perturbation to the network. For each secondary perturbations, amelioration or exacerbation of the network conversion risk is observed. For a detailed description of each secondary perturbation, see Section 2.4.1.

Using the initial monotonic portion of the function relating the perturbation of introducing bidirectional connections at different fractions to the rate of conversion of the network to an unhealthy HD-like dynamic (Figure 5, up to the 0.05 bidirectional fraction, 100% risk of conversion), we were able to also study risk to the network as a measure of this function. Specifically, we interpreted slope of this portion as a measure of risk, and went on to study how secondary perturbations to the network changed this slope (risk). Here we show how secondary perturbations such as changes to synaptic transmission ameliorated or exacerbated risks. In this way, ameliorating perturbations could be identified as therapeutic to the network dynamics we simulated. Specifically we created probability functions over the fraction of bidirectional connections in the presence of 5 different secondary perturbations. For each of these secondary perturbation simulations, bidirectional connections were added with *w*_*ij*_ = 0.01. We ran 20 simulations to compute the probability of an unhealthy network for each secondary perturbation, wherein each set of 20 is represented in Figure 7 by a single data point at the probability, for a total of 500 simulations.

Ameliorating secondary perturbations included an overall increase in GABAergic transmission among MSNs by increasing all weights (including newly added bidirectional connections) by 10% (Figure 7: GABA↑). This perturbation represents a homeostatic upregulation in transmission due to synaptic plasticity in the striatum (Cepeda et al., 2013).

Second, we modeled the same increase coupled with an increase of 10% in cortical excitatory input to each neuron responsible for the unhealthy dynamics (Figure 7: GABA↑ and GLUT ↑). This perturbation approximates a normal and local homeostatic response to increased cortical inputs by astrocytic GABA release during increased glutamate reuptake by the same glial cells through the GLT1 glutamate transporter (Wojtowicz et al., 2013).

Third, we modeled an overall decrease in GABAergic transmission throughout the network, together with a total shutdown of cortical excitation to the neuron responsible for unhealthy dynamics (Figure 7: GABA↓ and GLUT_OFF_). This perturbation approximated the condition in which GABA release from glial cells becomes widespread due to continuing excess glutamate release, causing a further desensitization of glutamate receptors on MSNs.

Each of these perturbations added to an overall decrease in the risk profile below baseline (Figure 7: Baseline), which we took to represent the WT condition.

Finally, we modeled one perturbation which exacerbated the risk function, namely an overall decrease in GABAergic transmission throughout the network (Figure 7: GABA↓) which approximates an overall decrease in glial cell GABA release (Estrada-Sanchez and Rebec, 2012).

#### 2.4.2. Bidirectional elimination by ongoing iSTDP

The previous studies aimed to examine perturbations to a healthy network dynamics by altering the stationary topology and homeostatic influences on the winnerless striatal network that brought the network initially to its healthy state. This topology was initialized with a random set of unidirectional connections between MSNs, then further refined with iSTDP.

**Figure 8:**
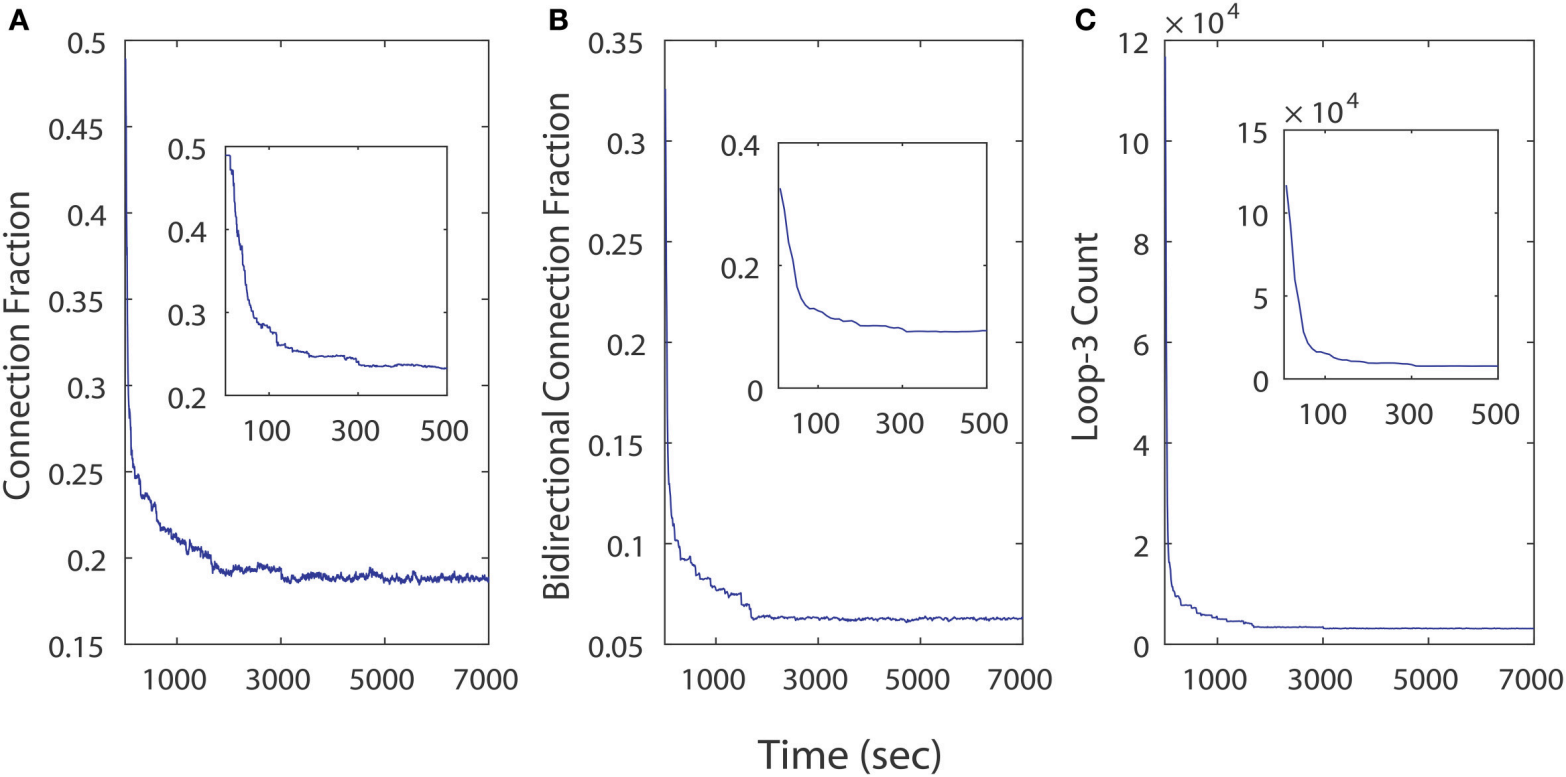
Topological changes in a 500 neuron network across time as a results of iSTDP. **(A)** Connection fraction decreases. **(B)** Bidirectional connection fraction decreases. **(C)** Loops of length 3 decrease in frequency.

We examined the influence of iSTDP in a network that is initialized with a very high connection fraction (~ 49%, Figure 8A) and bidirectional fraction (~ 32.5%, Figure 8B), which according to the previous results, in most instances will result in an unhealthy dynamic. Despite this, continuously computed iSTDP in this experiment was successful at reorganizing the network topology and radically reducing bidirectional connections, as well as loops of length three (Figure 8C) after 7, 000 simulated seconds. These results are consistent with n-order elimination of loops in recurrent excitatory network reported by Kozloski and Cecchi (2010). Specifically, the connection fraction converged to ~ 19%, which approximates the realistically observed fraction of connected pairs in the WT animal (Cepeda et al., 2013). Bidirectional connection fractions decreased to ~ 6% of connected pairs in this experiment with high initial connection fraction. In the stationary topology used for our perturbation studies, when bidirectional connections were added at a fixed and constant weight, this same fraction caused the network to become unhealthy (Figure 5). We observe therefore that ongoing iSTDP has complex influence on the network, and can accommodate higher rates of bidirectional connectivity while neutralizing the risk of conversion of network dynamics.

## 3. Discussion

Our results based on additions of homeostatic plasticity mechanisms to a winnerless network for establishing network health are consistent with the experimental findings of Cao et al. (2014), which showed that activity-dependent homeostatic regulation of excitability in MSNs is associated with enhancement of the M current. The M current is mediated by the voltage-gated potassium channel KCNQ, and is depressed in MSNs in the R6/2 HD mouse model (Cao et al., 2014). Both M current and the motor deficits that are observed concomitantly with changes in this current were partially reversed by acute exposure to M current activators. Therefore, our modeling result is well validated by these experimental findings, and elucidates possible mechanisms by which IP might maintain and restore healthy striatal network dynamics in the R6/2 mouse model.

We note that small fractions of cell silencing (i.e., < 2−3%) in the larger networks we studied failed to reliably produce unhealthy dynamics (Figure 3). The probability of developing unhealthy dynamics then increases rapidly as the fraction of neuron silencing increases from 0.2 to 0.6, before plateauing at 1. In this way, the putative effect of different stages of HD progression on striatal network dynamics, and potentially motor disturbances observed in HD in humans, are approximated and predicted by our model.

Comparing different curves in Figure 4 (inset), the weight of the added bidirectional connections is not monotonically related to the probability of developing unhealthy dynamics. In other words, while adding bidirectional connections to almost all networks increases the risk of unhealthy network dynamics, increasing the weight of the few added connections may increase or decrease this risk. For the highest weight (*w*_*ij*_ = 0.07), fluctuations in risk as the bidirectional fraction increased from 0.1 TO 0.4 occurred due to a driving influence on the network dynamics, by which the new weight dominated more than the previously learned weights (Figure 4). We also note that the lowest bidirectional fraction (0.001) approximates what should occur naturally in microcircuits undergoing structural plasticity, in which synapses are added spontaneously and at a low weight. The measured risk indicates that structural plasticity, in which new connections are introduced randomly at a low rate, within a winnerless network such as striatum should be harmless to the network dynamics.

In real neural systems, feedback from the the activity of the network onto iSTDP at synapses is ongoing, and can therefore compensate for the response to the perturbations we studied in real time. For example, we have already described how a low rate of structural plasticity (for purposes not explored in this paper) produces a minimal perturbation and little risk of an unhealthy dynamic. Continued structural plasticity without a regulating mechanism such as iSTDP, however, could potentially increase the bidirectional fraction leading to an unhealthy dynamic. Any absolute risk profile associated with these perturbations should be assumed to be magnified here by the absence of ongoing iSTDP. Since the sensitivity to the various perturbations we studied would likely be compensated by ongoing iSTDP, our predictions regarding real biological risks in striatum to the various perturbations relate only to their relative measures of risk.

The age of onset of Huntington's disease correlates with the number of poly-Q repeats beyond 37, with repeat lengths of 37–39 typically leading to emergence of the disease phenotype in a patient's sixties, while repeat lengths of 45–50 resulting in disease onset in a patient's thirties. Rarer, very long repeat lengths may cause phenotypic conversion in a patient's twenties or even as a juvenile. Typically, disease progression is modeled against a normalized “CAP” score (Ross et al., 2014), representing a patient's poly-Q length multiplied by age. This regularization of the main axis of disease progression is useful to compare across patients, but may mask underlying and different progression pathways that may be correlated to the range of Huntingtin protein poly-Q expansions.

In our study we observed several perturbations leading to an increased risk of a HD network phenotype in striatum, namely continuous activity in a small population of MSNs in an otherwise winnerless network. The dose-dependence of this risk on bidirectional connectivity (Figure 5) and on neuron silencing (Figure 3) presents an interesting opportunity to model disease progression pathways differently for different patient populations possessing different poly-Q lengths.

If, for example, poly-Q length is associated with a dose-dependent modification to plasticity between MSNs, as many studies of HD model animals suggest, the rate of bidirectional connectivity may be correlated with this change in the Huntingtin protein's structure. Neuron silencing in our model magnified the risk of further unhealthy dynamics, making it a potential strong feed forward influence in disease progression. Given these combined non-linearities, we predict that different ranges of poly-Q lengths may correlate with differently shaped non-linear disease progression functions (e.g., sigmoids with different slopes, Figure 3). It will be interesting to examine patient symptom progression such as motor dysfunction in those most likely to be affected by an unhealthy striatal dynamics.

## 4. Materials and methods

The winnerless network is composed of spiking FN neurons with the inhibitory interactions, described by first-order ordinary differential equations (Rabinovich et al., 2001),

(1) $$\begin{array}{r} \tau_{1}\frac{\text{d}x_{i}\left(t\right)}{\text{d}t}=f\left(x_{i}\left(t\right)\right)-y_{i}\left(t\right)-z_{i}\left(t\right)\left(x_{i}\left(t\right)+1.5\right)+r_{i}+\Theta_{i},\\ \frac{\text{d}y_{i}\left(t\right)}{\text{d}t}=x_{i}\left(t\right)-0.8y_{i}\left(t\right)+0.7,\\ \tau_{2}\frac{\text{d}z_{i}\left(t\right)}{\text{d}t}=\underset{j}{\sum }w_{ij}G\left(x_{j}\left(t\right)\right)-z_{i}\left(t\right),j\in \left[1,n\right], \end{array}$$

where *n* is the number of the neurons, *x*_*i*_(*t*) denotes the membrane potential, *y*_*i*_(*t*) is the recovery factor, *z*_*i*_(*t*) is the synaptic conductance modeled by first-order kinetics, *r*_*i*_ is the cortical stimulus, the elements of which are uniformly distributed within [0.2, 0.5], Θ_*i*_ regulates the neuron's excitability, $f\left(x\right)=x-\frac{1}{3}x^{3}$ represents the internal neuronal non-linearity, *G*(*x*) is a heaviside step function which gates the synaptic connection, *w*_*ij*_ denotes the inhibitory weights from neuron *j* to *i*. We set τ_1_ = 0.1 and τ_2_ = 10.

Due to the burst and bout nature of winnerless unit firing, neuronal plasticities are implemented in a discontinuous manner, with plastic changes implemented every 500 ms. To accomplish this, we divided the neuronal activity into 500 ms bins. For each bin, the neuron is considered active if a burst potential (FN spike) is observed. For the different forms of plasticity modeled in our study, this set of binary states (active/inactive), one for each 500 ms bin, provides input to our plasticity functions.

The *intrinsic plasticity* function regulates the neuron's excitability (Cao et al., 2014). To accomplish this, when a neuron is active, excitability is gradually lowered, while an inactive neuron's excitability is gradually increased. Both the decrease and increase are accomplished by shifting the threshold Θ_*i*_ using the same rate of change parameter η_*IP*_ = 0.001.

The *inhibitory spike-timing dependent plasticity (iSTDP)* function adjusts the synaptic weight *w*_*ij*_ logically to ensure inhibition is balanced against other drives of neuronal activation, such as the amount of cortical excitatory drive. To accomplish this, our logical criteria state that if the presynaptic neuron is active and, during the subsequent time bin, the postsynaptic neuron is inactive (i.e., the inhibitory presynaptic activity was “successful” in preventing the postsynaptic burst), *w*_*ij*_ is reduced by an amount η_*LTD*_ = 0.001. If after this reduction, *w*_*ij*_ < 0, the weight is set to a small value (0.001). If, however, a presynaptic neuron is active and in the subsequent time bin, the postsynaptic neuron is also active (i.e., the inhibitory spike was “unsuccessful” in preventing the the postsynaptic burst), the inhibitory weight is increased by the amount η_*LTP*_ = 0.01. Following these adjustments, weights were normalized, such that sum of all incoming weights was 4.

Studying causes of the unhealthy network dynamics described required a quantitative means to assign a given simulation to either the healthy or unhealthy class of dynamics. We determined this classification according to the following criterion. For each neuron in the network, if a neuron is active within 80% or more of all bins, the entire network was labeled unhealthy. Those neurons which met this criterion, were also deemed “responsible” for the unhealthy dynamics.

The single random network used for all perturbation experiments was evolved using iSTDP to create a healthy dynamic, after first confirming that for different other randomly generated networks, this method of initialization was robust. Random selection of neurons for silencing and random selection of neuron pairs for the introduction of a bidirectional connections were accomplished using MATLAB's random number generator to select the appropriate index, reseeded for each simulation using MATLAB's internal reseeding function. In all measures, fraction of neurons silenced is computed by the number of neurons silenced divided by the total number of neurons in the original network. Bidirectional connection fraction is defined as the number of pairs of neurons that have bidirectional connection divided by the number of pairs of neurons that originally had a unidirectional connection.

## Author contributions

PZ performed all simulations and analyses and contributed to the experimental design. JK supervised the work, wrote the manuscript, designed the model-based approach to network phenotypes of Huntington's disease, performed early simulations of the unhealthy network phenotype, and contributed to the experimental design.

### Conflict of interest statement

PZ and JK, are employees of the IBM Corporation or were employees at the time this research was conducted.

## Footnotes

^1^Each of these measures saw a decrease among the R6/2 HD model animals.
